# Integrated Analysis of Immune-Related Genes Reveals Altered Biological Functions and Immune Cell Abundance in Sleep Deprivation

**DOI:** 10.2174/0118715303444083260325043026

**Published:** 2026-05-07

**Authors:** Xiang Li, Xiaoyan Wang, Xiaodi Wang, Dan Bing

**Affiliations:** 1 Department of Otorhinolaryngology Head and Neck Surgery, Tongji Hospital Affiliated to Tongji Medical College of Huazhong University of Science & Technology, Hubei, Wuhan, China;; 2 Department of Immunology, School of Basic Medicine, Tongji Medical College, Huazhong University of Science & Technology, Hubei, Wuhan, China;; 3 Institute of Pathology, Tongji Hospital, Tongji Medical College, Huazhong University of Science and Technology, Wuhan, China

**Keywords:** Sleep deprivation, immune-related genes, immune cell composition, gene expression profiling, molecular subtype, gene expression omnibus

## Abstract

**Introduction:**

Sleep deprivation is a common phenomenon in modern society and has received widespread attention. However, data about its impact on immune system functions remains scant.

**Methods:**

Three sleep deprivation-related gene datasets, GSE98564, GSE98565, and GSE98566, were downloaded from the Gene Expression Omnibus database. A total of 23 control samples and 30 sleep deprivation samples were obtained after sample processing. Differentially Expressed Genes (DEGs) and Differentially Expressed Immune-Related Genes (DEIRGs) were analyzed with Gene Ontology and Kyoto Encyclopedia of Genes and Genomes enrichment analysis. Additionally, CIBERSORT analysis, weighted gene co-expression network analysis, and consensus clustering were performed. Finally, qPCR was conducted to verify the expression of six hub immune genes in the auditory cortex, hippocampus, and hypothalamus tissues from control and sleep-deprived mice.

**Results:**

The identified DEGs and DEIRGs affected immune and hematopoietic functions. The composition of neutrophils was significantly lower in the sleep deprivation samples than in the control group. Moreover, 32 key immune genes and five hub immune genes (HLA-DRB5, CTSE, ADIPOR1, HDGF, and ABCC4) associated with sleep deprivation were screened, which were also found to be significantly correlated with the proportions of major immune cells (naïve B cells, plasma cells, and neutrophils). Two molecular subtypes of sleep deprivation were identified, each with distinct immune genes, functions, pathways, and major immune cell composition.

**Discussion:**

Overall, the findings support the notion that sleep deprivation disrupts immune and hematopoietic pathways and alters immune-cell composition (notably reduced neutrophils with increased B-cell subsets), with hub immune genes and molecular subtypes suggesting biological heterogeneity that may inform future mechanistic and therapeutic studies. Furthermore, the function of these five hub immune genes should be investigated in both *in vitro* and *in vivo* models.

**Conclusion:**

The data from the study demonstrated that sleep deprivation affected the immune function and immune cell composition, especially neutrophil-mediated immune responses. The study reveals the underlying mechanisms of sleep deprivation-related immune disorders and offers potential treatment targets.

## 1 INTRODUCTION

Sleep deprivation, defined as a condition of insufficient or disrupted sleep, is an increasingly prevalent concern in modern societies due to lifestyle pressures, occupational demands, and prevalent sleep disorders [1]. Accumulating evidence links chronic sleep deprivation to a wide spectrum of adverse health outcomes, including heightened risks for neuropsychiatric disorders (*e.g*., depression), metabolic dysregulation (such as diabetes), and exacerbated inflammatory and oxidative stress responses [2-4]. Moreover, prolonged lack of adequate sleep has been found to disrupt the functioning of the nervous, endocrine, and immune systems, thus predisposing individuals to various infectious and non-infectious diseases [5-7].

Despite growing recognition of the broad health impacts of sleep deprivation, our understanding of how it disrupts immune system function at the molecular and cellular levels remains limited. Prior studies have illustrated that sleep plays a critical role in regulating both adaptive and innate immunity. For instance, sleep restriction has been associated with alterations in cytokine profiles—namely, increased levels of the anti-inflammatory cytokine IL-10, which favors a Th2-skewed immune response, and decreased production of IL-12, essential for Th1 response modulation [8, 9]. In individuals with chronic sleep loss, marked reductions in lymphocyte subsets, including CD3+, CD4+, and CD8+ T cells, have been reported [10]. Similarly, experimental sleep deprivation in humans and animal models leads to suppressed Natural Killer (NK) cell activity and elevated circulating inflammatory mediators such as Tumor Necrosis Factor (TNF) and Interleukin-6 (IL-6), further suggesting broad immunological consequences of sleep loss [11-13].

While these studies have established a link between sleep deprivation and immune dysregulation, the precise molecular mechanisms and the landscape of immune gene expression alterations remain incompletely characterized. The complexity and heterogeneity of immune responses under sleep deprivation highlight the need for comprehensive approaches that can integrate gene expression data with immune cell profiling. Recent advances in high-throughput transcriptomics and systems biology methods, such as Weighted Gene Co-expression Network Analysis (WGCNA), have facilitated the identification of critical regulatory genes and molecular networks underlying complex diseases [14-17]. WGCNA, in particular, enables the delineation of gene modules that are highly correlated with specific clinical traits, thus offering insights into key drivers of disease phenotypes. Additionally, developments in molecular subtyping based on transcriptomic data have proven invaluable for revealing disease heterogeneity, guiding biomarker discovery, and informing personalized therapeutic strategies in various conditions [18, 19]. However, to date, few studies have systematically applied such integrative molecular approaches to characterize immune-related alterations and molecular subtypes in sleep deprivation.

Addressing these gaps, the present study leverages three publicly available sleep deprivation-related gene expression datasets to conduct an integrated analysis of Differentially Expressed Genes (DEGs) and Immune-Related Genes (DEIRGs) between sleep-deprived and control samples. By employing functional enrichment analyses (Gene Ontology and KEGG), WGCNA, immune cell deconvolution (CIBERSORT), and consensus molecular subtyping, we aim to elucidate the altered biological pathways and immune cell landscape associated with sleep deprivation. Furthermore, we validate key hub immune genes in the auditory cortex, hippocampus, and hypothalamus tissues using quantitative PCR. This comprehensive approach seeks to advance our understanding of the immunological mechanisms underlying sleep deprivation, identify potential biomarkers, and lay the groundwork for targeted interventions to mitigate sleep deprivation-induced immune dysfunction.

## 2 MATERIALS AND METHODS

### 2.1 Study Design and Type of Study

This study employed an integrated translational design combining retrospective bioinformatics analysis with prospective animal validation to investigate immune-related molecular alterations in sleep deprivation. The research comprised two distinct phases:

Phase 1 (Retrospective *in silico* analysis): A secondary analysis of publicly available human transcriptomic data was conducted to identify immune-related biomarkers and molecular subtypes associated with sleep deprivation. This phase utilized three microarray datasets (GSE98564, GSE98565, GSE98566) from the Gene Expression Omnibus (GEO) database, comprising 163 control and sleep-deprived individuals. The bioinformatics pipeline included: (a) identification of Differentially Expressed Genes (DEGs) and Immune-Related Genes (IRGs), (b) Weighted Gene Co-expression Network Analysis (WGCNA) to discover hub immune genes, (c) unsupervised clustering to define molecular subtypes, and (d) *in silico* immune cell deconvolution.

Phase 2 (Prospective animal validation): Validatory experiments were performed using a controlled laboratory animal model to corroborate bioinformatics findings and assess tissue-specific expression of candidate genes. This phase employed a parallel-group, controlled experimental design in C57BL/6J male mice (n = 6/group) subjected to 22-hour daily sleep deprivation for 21 consecutive days.

The integration of these complementary approaches enables cross-species validation of molecular mechanisms and provides translational relevance to the human transcriptomic findings. The workflow proceeded from discovery (human data) to validation (animal model), establishing a robust framework for identifying clinically actionable immune-related targets in sleep deprivation.

### 2.2 Data Acquisition and Pre-processing

Raw CEL files for GSE98564, GSE98565, and GSE98566 were retrieved from the Gene Expression Omnibus (GEO) database [20]. These three datasets were selected because they were generated by the same research group using a highly consistent experimental design (peripheral blood sampling under a comparable sleep-deprivation protocol and time-point setting) and the same microarray platform (GPL6244), which reduces technical heterogeneity and facilitates reliable cross-study integration. In the initial study design, we also screened GEO for additional human peripheral-blood transcriptome datasets related to sleep deprivation; however, most available datasets were not suitable for integrative analysis due to limited sample size, substantial platform differences, and/or incomplete experimental designs (*e.g*., lack of an appropriate control condition). Therefore, we focused on GSE98564–66 as the most methodologically homogeneous and sufficiently powered resources for combined analysis.

All preprocessing was conducted using the oligo package [21]. Array quality was evaluated using array-intensity distributions and RNA degradation plots. Background correction, probe summarization, and quantile normalization were performed using the Robust Multiarray Average (RMA) procedure. Probes were mapped to gene symbols using the platform annotation; when multiple probes mapped to the same gene, their expression values were aggregated by averaging to generate a gene-level expression matrix. To minimize cross-dataset measurement differences, only genes shared across all three datasets were retained prior to merging.

Because the combined cohort originated from multiple GEO series, potential batch effects attributable to the dataset source were explicitly addressed. After merging, batch effects were corrected using the ComBat function in the sva package with GEO series as the batch variable, followed by between-array normalization using normalizeBetweenArrays in the limma package to ensure comparable expression distributions across samples. After these steps, principal component analysis did not indicate systematic clustering by dataset, supporting the adequacy of batch handling for downstream differential expression, network analysis, and immune deconvolution.

After excluding fatigue-resistant samples as defined in the original study, technical replicates were averaged. Two outlier samples identified by principal component analysis were removed. The final analytic cohort included 53 samples (23 controls and 30 sleep-deprived). Normalization performance was assessed using boxplots of normalized intensities (random subset of samples), and dataset characteristics are summarized in Table **S1**.

### 2.3 Identification and Enrichment Analysis of DEGs

Using the microarray datasets, DEGs were identified with the limma package [22] for the gene expression matrix after pre-processing, with the threshold of adjusted p-value (adj.p) < 0.05 and |log2FC| > 1. The DEG profile was plotted using the ggplot2 package for volcano maps and the pheatmap package for heat maps. ClusterProfiler package [23] was then utilized to perform function and pathway enrichment analyses for the DEGs and Gene Set Enrichment Analysis (GSEA) of all genes. Entries with p-value < 0.05 were considered significant enrichment, and the top 20 were visualized as bubble plots.

### 2.4 Assessment of the Immune Cell Abundances

The CIBERSORT algorithm was used to determine the abundance of 22 immune cells. CIBERSORT is a deconvolution algorithm for a gene expression matrix that estimates the composition and abundance of immune cells in a mixture of cells [24]. The ggpubr package was used to visualize differences in the abundance of 22 immune cells between the sleep deprivation and control samples. Correlation between key immune genes and major immune cell abundances was also plotted.

### 2.5 Screening and Enrichment Analysis DEIRGs

The Immune-Related Genes (IRGs) were downloaded from the ImmPort (https://immport.niaid.nih.gov) database [25]. Overlapping genes between the IRGs and DEGs were considered to be the DEIRGs. The functions and expression of DEIRGs were analyzed by the Metascape database [26] and visualized by boxplots, respectively.

### 2.6 Analysis of WGCNA and Selection of Hub Genes

The weighted gene co-expression network was constructed using the WGCNA package [17]. By using an appropriate power of β for the Pearson correlation coefficient between gene expression values, the WGCNA makes the constructed network more compatible with scale-free network criteria. The adjacency matrix was transformed into a topological overlap matrix. To delineate functional modules, hierarchical clustering was performed based on the similarity of the expressed genes.

### 2.7 Identification of Key Immune Genes and Hub Immune Genes

Genes from the modules that were most relevant to the clinical sleep deprivation phenotype were extracted and intersected with the IRGs. Overlapping genes were considered key immune genes, and their functions were predicted on the Metascape database [26]. Additionally, overlapping genes between key immune genes and DEGs were also considered as hub immune genes.

### 2.8 Identification of Sleep Deprivation Molecular Subtypes

The molecular subtypes of sleep deprivation were calculated using the ConsensusClusterPlus package [27], based on the most appropriate k-values. The subtypes were visualized through dimensionality reduction using the Rtsne package. Differences in hub gene expression and immune cell composition between the molecular subtypes were plotted using the ggpubr package.

Furthermore, DEGs between the two molecular subtypes were analyzed using the limma package based on the threshold of adj. p < 0.05 and |log2FC| > 1. The ClusterProfiler package was used to perform function and pathway enrichment analysis of the DEGs and Gene Set Enrichment Analysis (GSEA) of all genes. Entries with p-value < 0.05 were considered to be significantly enriched, among which the top 20 were visualized as bubble plots.

### 2.9 Animal Experiments of Sleep Deprivation Models

Adult C57BL/6J male mice (8 weeks old, weight 20–22 grams) were procured from the Model Animal Research Center of Tongji Medical College of Huazhong University of Science and Technology (Wuhan, China). Upon arrival, animals were housed individually in standard Plexiglas cages (30 × 15 × 12 cm) within a Specific Pathogen Free (SPF) facility. Husbandry conditions were strictly controlled: temperature 22 ± 2°C, relative humidity 50–60%, and a 12:12 hour light/dark cycle. All mice had ad libitum access to standard laboratory rodent chow and filtered water. Cages were lined with corncob bedding replaced weekly, and contained environmental enrichment (nesting material and a PVC tube). All animals were allowed a 7-day acclimation period before experimental manipulation to minimize transport stress and establish baseline circadian rhythms. The health status of each mouse was verified by a veterinary examination prior to study initiation. The experimental unit was one individual mouse.

Following acclimation, the animals were randomly assigned to the control and sleep-deprivation groups using a computer-generated random-number table. The sample size was determined based on previously published sleep deprivation studies in C57BL/6J mice employing comparable group sizes (n = 6 per group) to detect biologically meaningful effect sizes with adequate statistical power while minimizing animal use according to the 3Rs principle. This sample size was reviewed and approved by the Animal Research and Ethics Committee of Tongji Medical College, Huazhong University of Science and Technology (NO.: TJH-202006007), in compliance with the Chinese guidelines for the care and use of animals.

The sleep deprivation intervention was administered at a daily exposure of 22 hours (8:00 pm–6:00 pm) at a frequency of 7 days/week for three consecutive weeks (study period: September 2, 2022–January 13, 2026). The procedure was carried out using a specialized apparatus comprising a cylindrical vessel and console, with a rotating rod at the base controlled by the console. The rotating rod speed was maintained at 12 seconds per turn and operated intermittently in 15-second cycles (15 seconds of rotation followed by 15 seconds of intermission), preventing mice from achieving sustained sleep episodes through tactile stimulation. The control group was maintained under normal housing conditions without intervention throughout the study period.

 During the three-week study period, all mice were monitored twice daily (at 08:00 and 18:00) by trained technicians blinded to group allocation. Health records were maintained for each mouse, and any animal exhibiting signs of distress, injury, or illness beyond mild severity was reported immediately to the attending veterinarian. At the conclusion of the three-week deprivation period, mice were euthanized *via* inhalation of isoflurane (R510-22-10, RWD Life Science Co., Ltd.) followed by cervical dislocation to ensure death. All procedures were performed in a dedicated procedure room between 09:00 and 11:00 to minimize circadian variation. No animals reached humane endpoints or died prematurely during the study.

### 2.10 Quantitative Polymerase Chain Reaction (qPCR)

Auditory cortex, hippocampus, and hypothalamus tissues were collected, and total RNA was extracted using TRIzol Reagent following the manufacturer’s protocol. The extracted mRNA (1μg) served as a template to synthesize cDNA using the PrimerScript RT Master Mix kit (Takara, Dalian, China). Real-time PCR was performed on a LightCycler System 2.0 (Roche, Mannheim, Germany) using SYBR Premix EX Taq kit (Takara, Dalian, China). The RT-PCR was performed at 95°C for 5 min, then 95°C (45 seconds), 56°C (30 seconds), and 72°C (45 seconds) for 40 cycles, followed by a 10 minutes extension at 72°C. Each sample was run in triplicate. Gene expression was normalized to β-actin, and relative gene expression was calculated by the 2^-△△Ct^ method. The primer sequences used in the PCR were as follows: Abcc4 forward primer: 5’-GGCACTCCGGTTAAGTAACTC-3’, reverse primer: 5’-TGTCACTTGGTCGAATTTGTTCA-3’. Adipor1 forward primer: 5’-TCTTCGGGATGTTCTTCCTGG-3’, reverse primer: 5’-TTTGGAAAAAGTCCGAGAGACC-3’. CTSE forward primer: 5’-GACATCAGTCCCTTCGGAAGA-3’, reverse primer: 5’-AGGGGTTCATTGACACTCGAATA-3’. H2-Eb1 forward primer: 5’-GCGGAGAGTTGAGCCTACG-3’, reverse primer: 5’-CCAGGAGGTTGTGGTGTTCC-3’. H2-Eb2 forward primer: 5’-GTGTGTGGAGTGTGACCAGAT-3’, reverse primer: 5’-GGCACGATAATTGTCCAGCAT-3’. Hdgf forward primer: 5’-CCGGATTGATGAGATGCCTGA-3’, reverse primer: 5’-TTGCCAAACTTCTCCTTGGATT-3’. β-actin forward primer: 5’-GCGCAAGTACTCTGTGTGGA-3’, reverse primer: 5’- GAAAGGGTGTAAAACGCAGC-3’.

### 2.11 Statistical Analysis

All computational analyses were performed in R 4.2.1. Batch effects across datasets were corrected using ComBat-Seq. Multiple testing correction was applied using Benjamini-Hochberg False Discovery Rate (FDR).

qPCR results are presented as mean ± Standard Deviation (SD) of n = 6 biological replicates. Normality was tested using the Shapiro-Wilk test. Unpaired two-tailed Student's t-tests were used for normally distributed data with equal variance; Mann-Whitney U tests were used for non-parametric data. Statistical significance was set at *p* < 0.05. All graphs were generated using GraphPad Prism 9.0.

## 3 RESULTS

### 3.1 Data Pre-processing

**Fig. (1):**
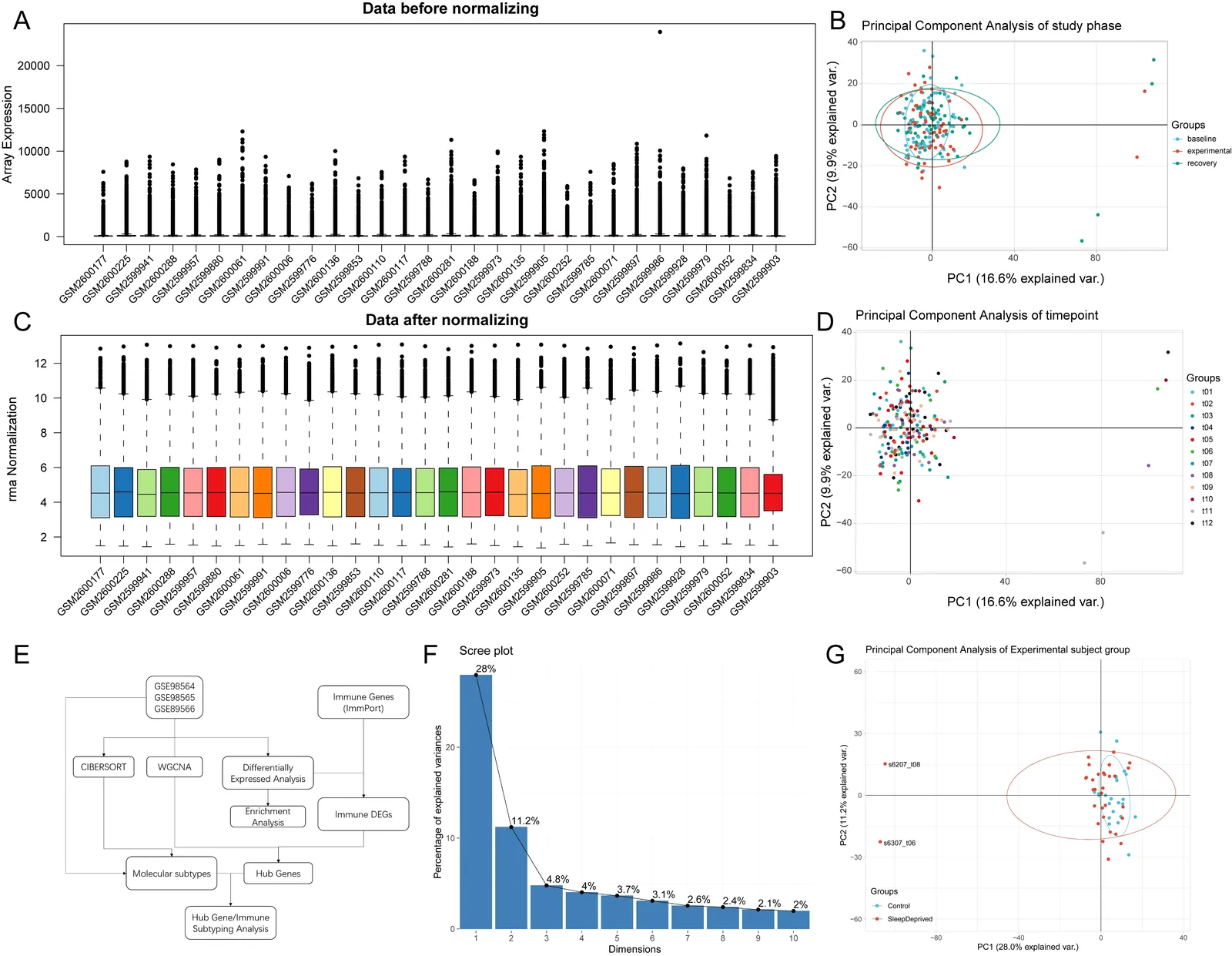
Pre-processing of sleep deprivation-related microarray datasets. GSE98564, GSE98565, and GSE98566 were included, and the samples were processed; 23 control samples and 30 sleep deprivation samples were obtained for further analysis. (**A**) Array expression before Robust Multiarray Average (RMA) normalization. (**B**) Principal Component Analysis (PCA) of all the samples grouped by study phase. (**C**) Array expression after RMA normalization. D, PCA of all samples grouped by time point. (**E**) The analysis workflow diagram. F, Scree plot of 55 samples after sample processing. (**G**) PCA plot of 55 samples after sample processing.

GSE98564, GSE98565, and GSE98566 microarray datasets were downloaded from the GEO database, comprising 555 samples (Fig. **1A**). Principal component analysis (PCA) was conducted to explore the distribution between groups (Fig. **1B**). The raw data were subjected to background correction and normalization using the RMA algorithm in the R language (Fig. **1C**, Fig. **1D**). Detailed workflow is shown in Fig. (**1E**). Considering that each sample in these datasets contained multiple biological replicates, the mean value of the expression profiles for each sample was used. Moreover, two outlier samples were identified and removed. Finally, 23 control samples and 30 sleep deprivation samples were obtained for further analysis (Fig. **1F** and **1G**).

### 3.2 Analysis of the DEGs

**Fig. (2):**
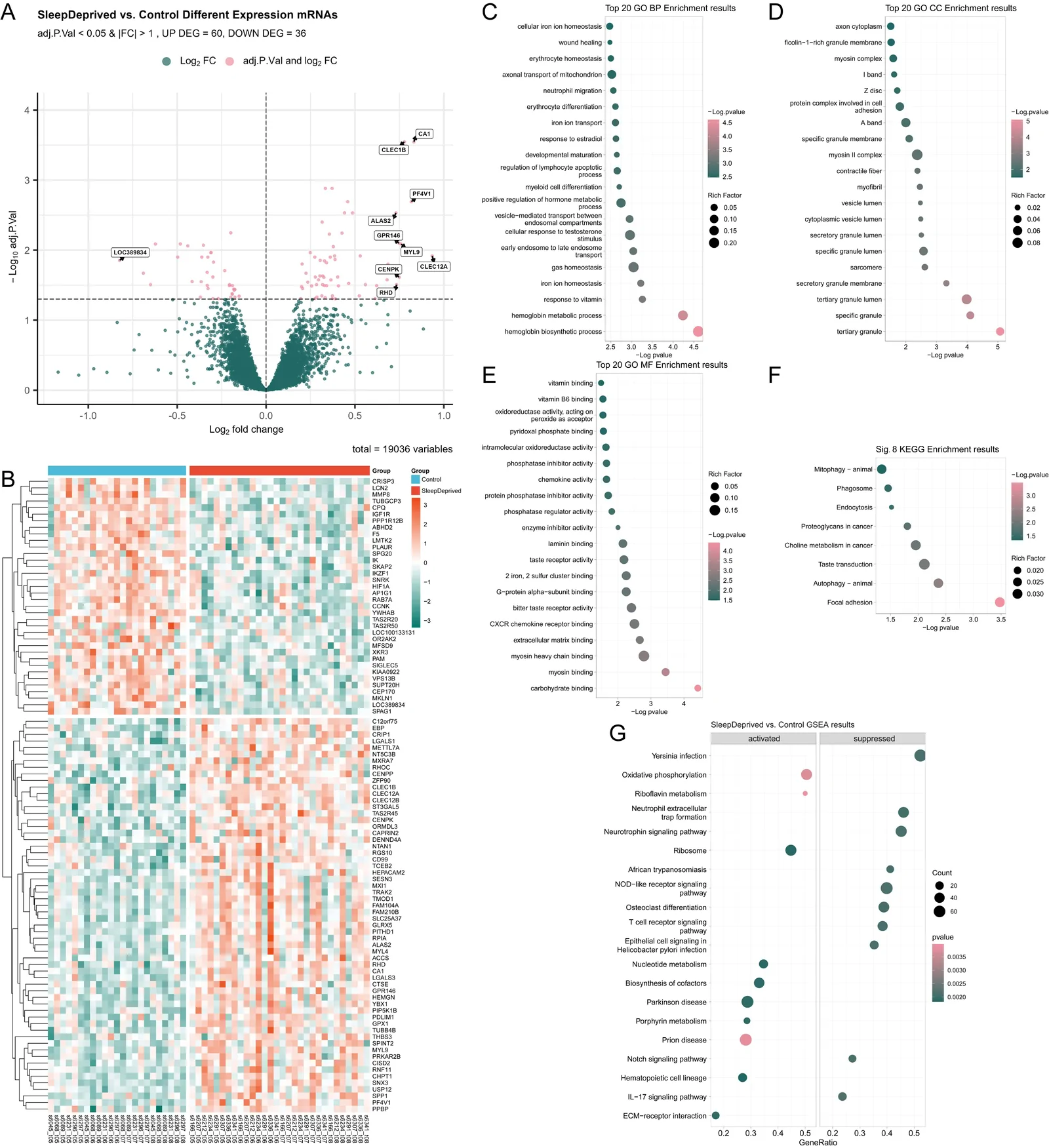
Identification of Differentially Expressed Genes (DEGs) and enrichment analysis. Gene expression volcano map (**A**) and heat map (**B**) of the control and sleep deprivation samples. Enriched Biological Process (BP) (**C**), cellular component (CC) (**D**), Molecular Function (MF) (**E**), and Kyoto Encyclopedia of Genes and Genomes (KEGG) (**F**) terms for DEGs. G, Gene Set Enrichment Analysis (GSEA) analysis showing pathways significantly related to sleep deprivation.

Differential analysis was performed on the processed samples and visualized in a volcano map and a heat map (Fig. **2A** and **B**). The data showed that 60 genes were up-regulated while 36 genes were down-regulated.

To identify the potential functions of the DEGs, GO enrichment analysis was conducted (Supplementary Table **S2**). The Biological Process (BP) enrichment results revealed that the DEGs were significantly enriched in cellular iron ion homeostasis, wound healing, erythrocyte homeostasis, axonal transport of mitochondrion, neutrophil migration, erythrocyte differentiation, iron ion transport, response to estradiol, developmental maturation, regulation of lymphocyte apoptotic process, myeloid cell differentiation, positive regulation of hormone metabolic process, vesicle-mediated transport between endosomal compartments, cellular response to testosterone stimulus, early endosome to late endosome transport, gas homeostasis, iron ion homeostasis, response to vitamin, hemoglobin metabolic process, and hemoglobin biosynthetic process, thus suggesting that sleep deprivation might have a significant impact on immune and hematopoietic functions (Fig. **2C**). The bubble diagram in Fig. (**2D** and **E**) shows the enriched Cellular Component (CC) and Molecular Function (MF) terms. Consistent with the previous GO analysis, the KEGG analysis (Fig. **2F**, Supplementary Table **S2**) and GSEA (Fig. **2G**, Supplementary Table **S2**) showed that the DEGs were involved in mitophagy-animal, phagosome, endocytosis, proteoglycans in cancer, choline metabolism in cancer, taste transduction, autophagy-animal, focal adhesion, yersinia infection, oxidative phosphorylation, riboflavin metabolism, neutrophil extracellular trap formation, neurotrophin signaling pathway, ribosome, African trypanosomiasis, NOD-like receptor signaling pathway, osteoclast differentiation, T cell receptor signaling pathway, epithelial cell signaling in helicobacter pylori infection, nucleotide metabolism, biosynthesis of cofactors, Parkinson disease, porphyrin metabolism, Prion disease, Notch signaling pathway, hematopoietic cell lineage, IL-17 signaling pathway, and ECM-receptor interaction.

### 3.3 Immune Cell Abundances Between the Sleep Deprivation and Control Samples and Analysis of the DEIRGs

**Fig. (3):**
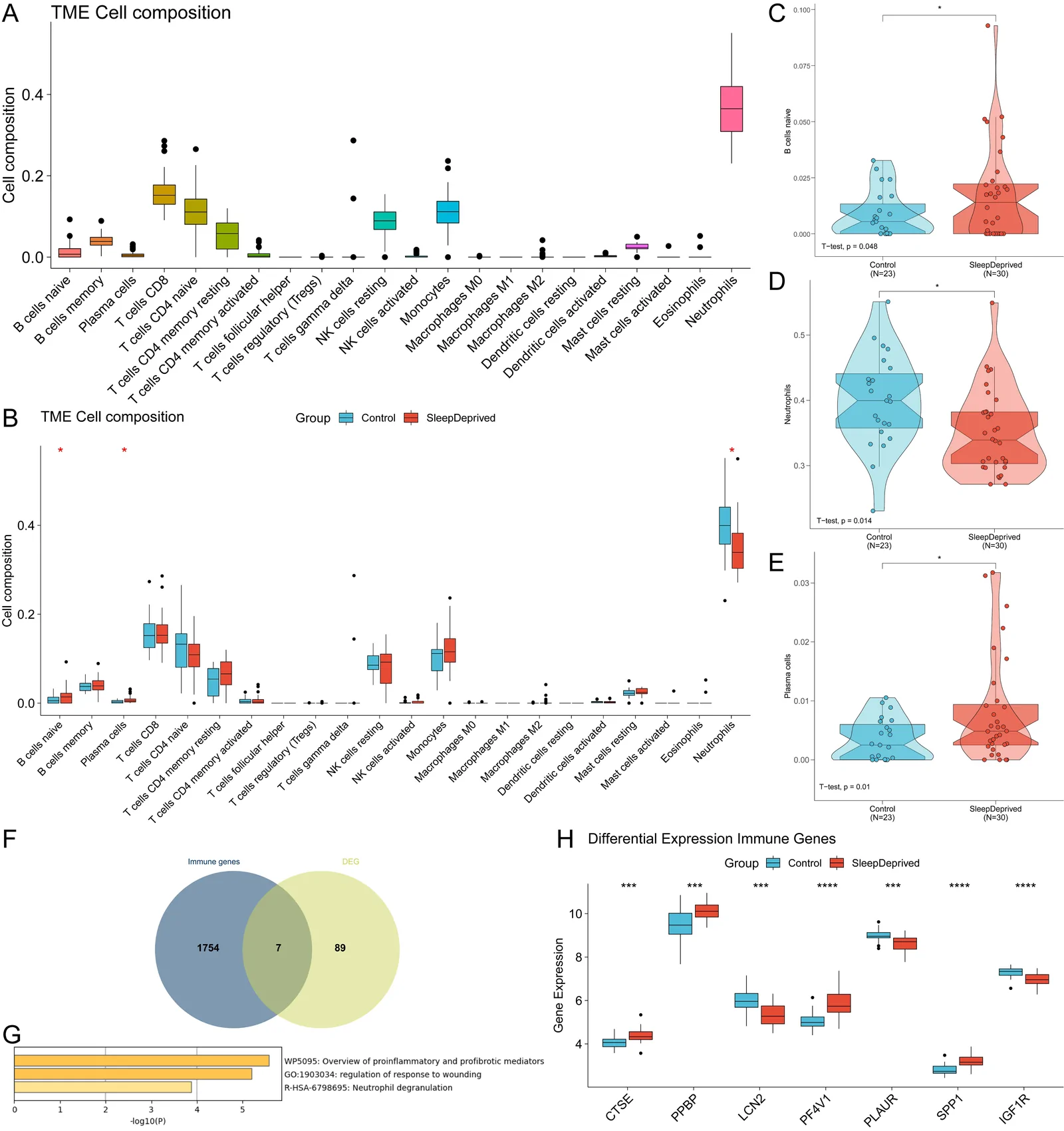
Sleep deprivation–associated alterations in immune cell composition and immune-related transcriptomic signatures. (**A** and **B**) Relative proportions of 22 immune cell types estimated by CIBERSORT in the control and Sleep Deprivation (SD) groups and between-group comparisons. Neutrophils were significantly decreased in SD samples, whereas naïve B cells and plasma cells were increased. (**C–E**) Distributions of the significantly altered immune cell types (naïve B cells, neutrophils, and plasma cells) between groups. (**F**) Venn diagram showing the overlap between Differentially Expressed Genes (DEGs) and ImmPort immune-related genes to identify Differentially Expressed Immune-related Genes (DEIRGs). (**G**) Functional enrichment analysis of DEIRGs, highlighting inflammatory responses and neutrophil-related processes. (**H**) Expression patterns of DEIRGs in the control and SD groups. *, *p* < 0.05; ***, *p* < 0.001; ****, *p* < 0.0001.

To understand differences in immune cell abundances between the sleep-deprivation and control samples, we estimated immune cell composition using the CIBERSORT package. The data showed that neutrophils were the main immune cells in the microenvironment and were significantly downregulated in sleep deprivation samples (Fig. **3A** and **3B**). In addition, naïve B cells and plasma cells were significantly upregulated in the sleep-deprivation samples (Fig. **3B**). We then explored the differences in naïve B cells, neutrophils, and plasma cells across the samples (Fig. **3C**-**3E**).

To obtain DEIRGs, we intersected the immune genes from the ImmPort database with DEGs (Fig. **3F**). The 7 overlapped genes were enriched in an overview of proinflammatory and profibrotic mediators, regulation of response to wounding, and neutrophil degranulation (Supplementary Table **S3**, Fig. **3G**). Among the 7 genes, CTSE, PPBP, PF4V1, and SPP1 were upregulated in sleep deprivation samples, while LCN2, PLAUR, and IGF1R were downregulated in sleep deprivation samples (Fig. **3H**).

### 3.4 Analysis of the WGCNA and Identification of Key Immune Genes

**Fig. (4):**
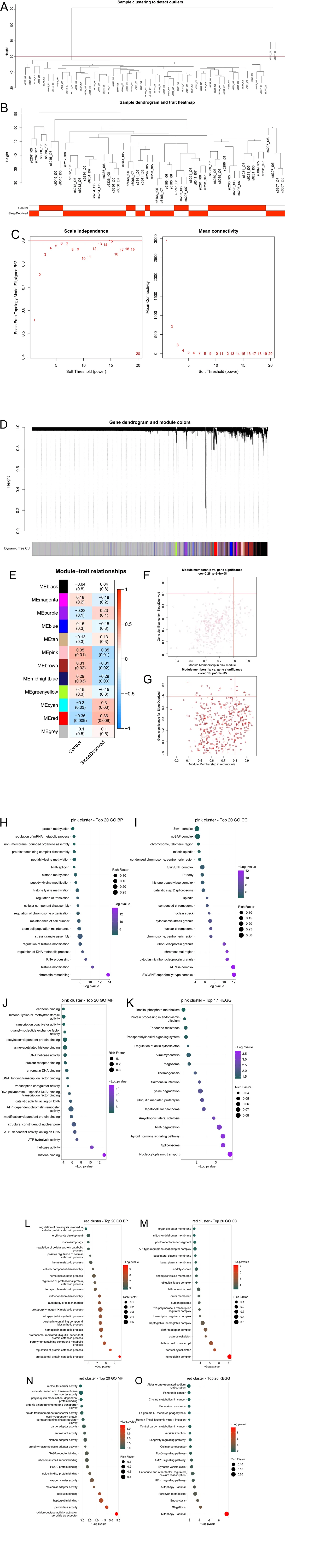
Construction of the co-expression network. (**A**) Sample clustering for detecting outliers. (**B**) Hierarchical clustering after removal of outliers. (**C**) The scale-independence index and mean connectivity for various soft threshold powers. (**D**) Co-expression gene modules detected by hierarchical cluster analysis. (**E**) Correlation between modules and sleep deprivation status, with orange indicating positive correlation and blue indicating a negative correlation. The darker the color, the stronger the correlation. (**F**) Correlation between Module Membership (MM) and Gene Significance (GS) in the pink module. (**G**) Correlation between Module Membership (MM) and Gene Significance (GS) in the red module. H-K, Enrichment analysis for genes in the pink module, including GO-BP enrichment (**H**), GO-CC enrichment (**I**), GO-MF enrichment (**J**), KEGG enrichment (**K**). **L-O**, Enrichment analysis for genes in the red module, including GO-BP enrichment (**L**), GO-CC enrichment (**M),** GO-MF enrichment (**N**), and KEGG enrichment (**O**).

To identify sleep deprivation-related genes, we performed Weighted Gene Co-expression Network Analysis (WGCNA) on the filtered samples. Sample clustering identified two outlier samples, which were subsequently removed (Fig. **4A** and **B**). To construct a network that approximated scale-free topology, we selected a soft threshold power of 15. This value was chosen because it yielded a scale-free topology fit index (R^2^) slightly above 0.9—the commonly accepted threshold for satisfactory scale-free network properties—as indicated by the red dashed line in Fig. (**4C**). Using the Dynamic Tree Cut algorithm, genes with similar expression patterns were clustered into modules, resulting in a total of 12 modules (Fig. **4D**). The pink and red modules showed the strongest association with sleep deprivation (Fig. **4E**). In both modules, a strong positive correlation was observed between Gene Significance (GS) and Module Membership (MM), indicating that hub genes in these modules are biologically important in the context of sleep deprivation (Figs. **4F** and **4G**). Genes within these two modules were then subjected to functional enrichment analysis for Biological Process (BP), Cellular Component (CC), Molecular Function (MF), and KEGG pathways (Fig. **4H**–**4O**).

**Fig. (5):**
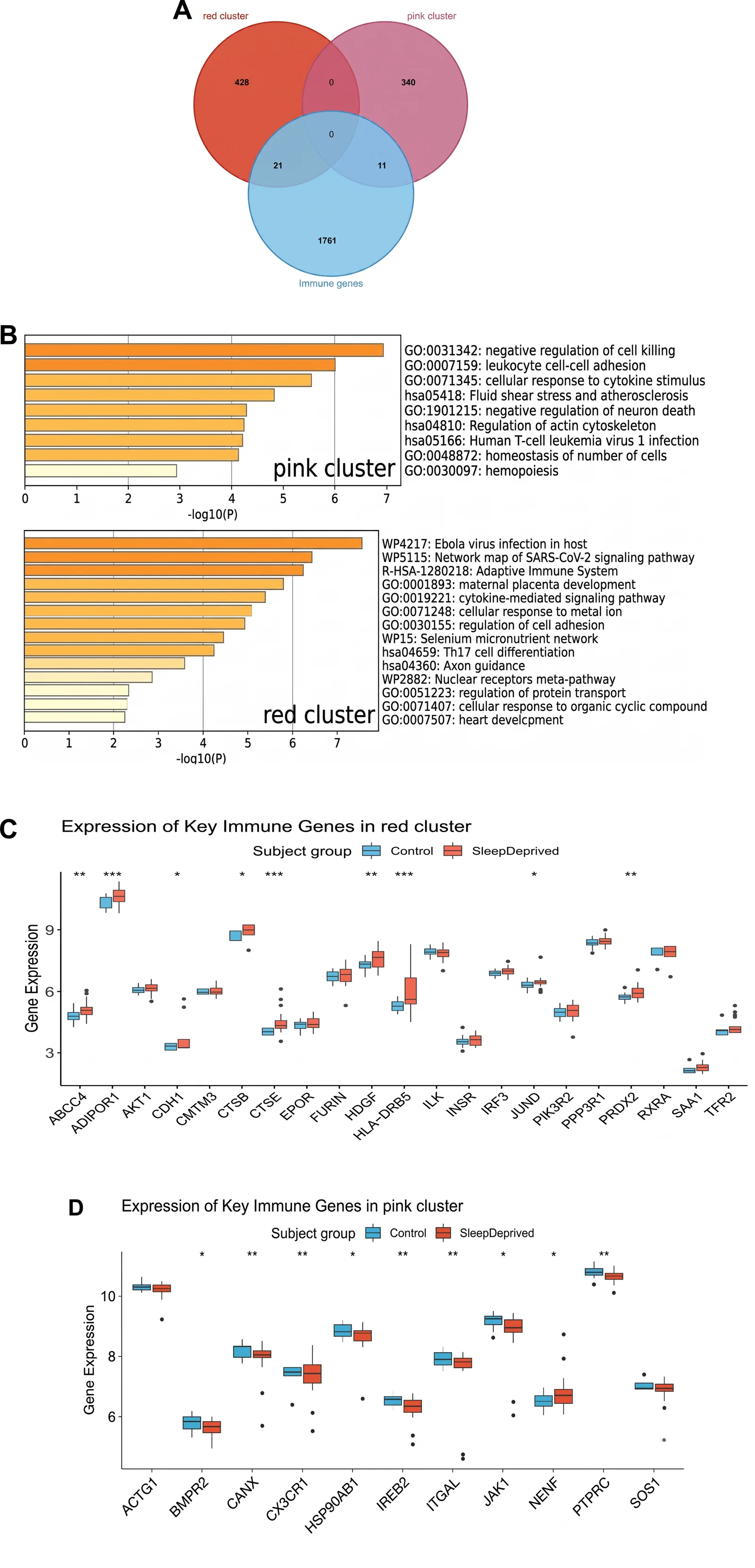
Identification and expression profiles of key immune genes associated with sleep deprivation. (**A**) Overlap between genes from SD-associated co-expression modules (pink and red modules) and immune-related genes to define key immune genes. (**B**) Functional enrichment analysis of key immune genes. (**C** and **D**) Expression distributions of key immune genes in the control and SD groups for the red module (**C**) and pink module (**D**). Several immune-related genes showed significant differential expression under SD, suggesting their potential involvement in SD-related immune regulation. *, *p* < 0.05; **, *p* < 0.01; ***, *p* < 0.001.

Subsequently, we intersected genes in these two modules and IRGs. The overlapping genes were considered as key immune genes (Fig. **5A**). Fig. (**5B**) shows the functions and pathways associated with the expression of overlapping genes (Fig. **5C** and **D**). Among key immune genes, HLA-DRB5, CTSE, ADIPOR1, HDGF, and ABCC4 were regarded as hub immune genes.

### 3.5 The Relationship Between Key Immune Genes and Immune Cells

**Fig. (6):**
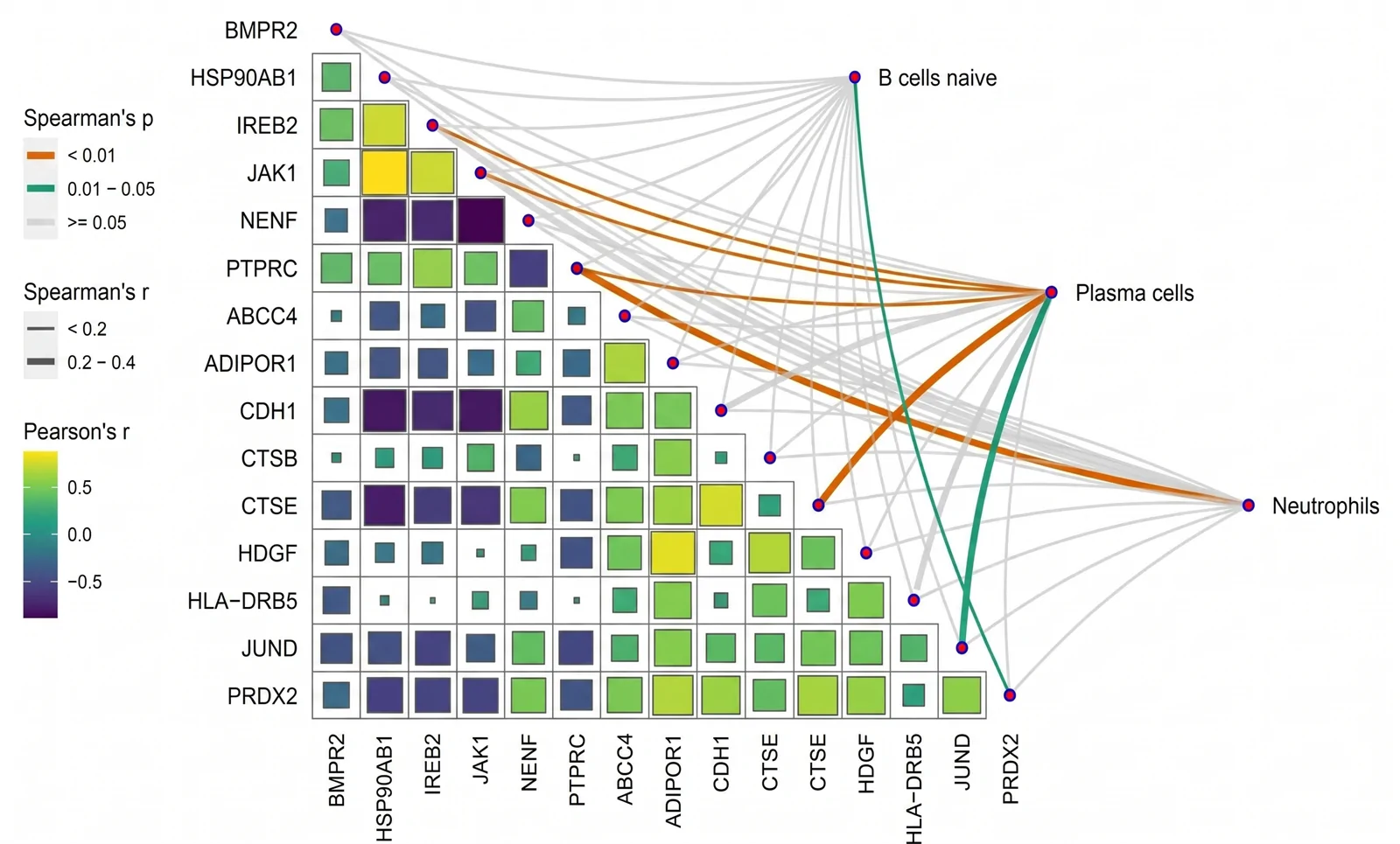
The correlation matrix of major immune cell abundances and key immune genes. The key immune genes with differential expression between sleep deprivation and control samples were selected and subjected to correlation analysis.

To investigate the correlation between the differential key immune genes and major immune cells, a correlation matrix was constructed. The abundance of naïve B cells was found to be positively correlated with PRDX2 expression. However, the abundance of plasma cells was positively correlated with IREB2, JAK1, PTPRC, CTSE, and JUND. Neutrophil abundance was positively correlated with PTPRC (Fig. **6**).

### 3.6 Identification of Sleep Deprivation Molecular Subtypes and Analysis of Their Characteristics

**Fig. (7):**
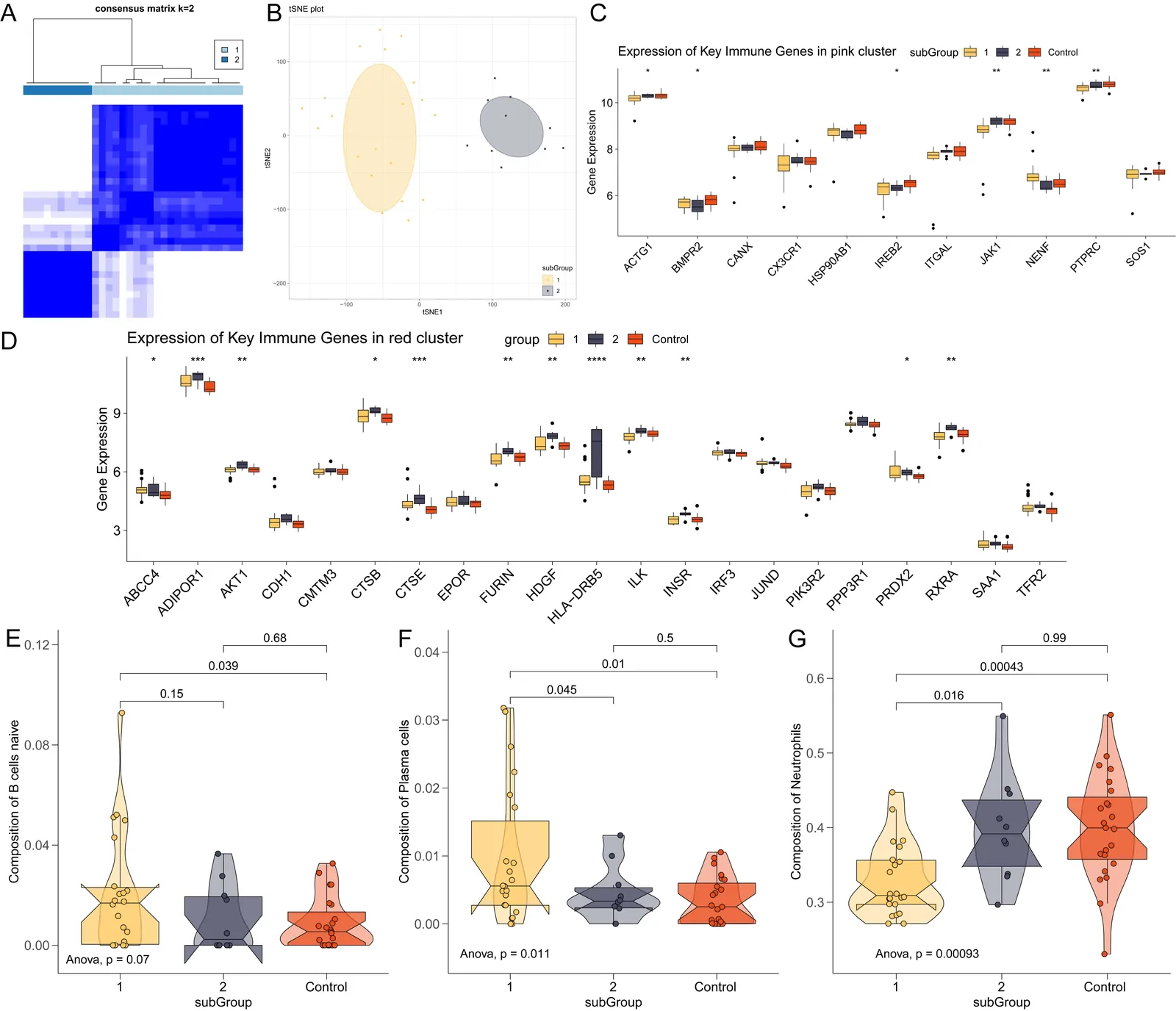
Molecular subtypes of sleep deprivation and their distinct immune features. (**A**) Consensus clustering identifies two SD subtypes (k = 2). (**B**) t-SNE visualization shows clear separation of the two subtypes based on gene expression profiles. (**C, D**) Differential expression of key immune genes among subtype 1, subtype 2, and control for the pink module (**C**) and red module (**D**). (**E–G**) Differences in major immune cell proportions among subtype 1, subtype 2, and control, including naïve B cells (E), plasma cells (**F**), and neutrophils (**G**). These results indicate heterogeneity in immune-related gene expression and immune cell composition across SD subtypes, implying distinct immune states in individuals with SD. *, *p* < 0.05; **, *p* < 0.01; ***, *p* < 0.001.

To further characterize sleep deprivation, we performed molecular subtyping of the sleep deprivation samples using the ConsensusClusterPlus package (Fig. **7A**). The samples were divided into two subtypes (Supplementary Table **S4**) and verified by t-SNE (Fig. **7B**). Interestingly, the key immune genes (Fig. **7C** and **D**) and major immune cells (Fig. **7E** - **G**) showed a distinct distribution in the molecular subtypes of sleep deprivation, suggesting that each subtype has a relatively different immune microenvironment.

**Fig. (8):**
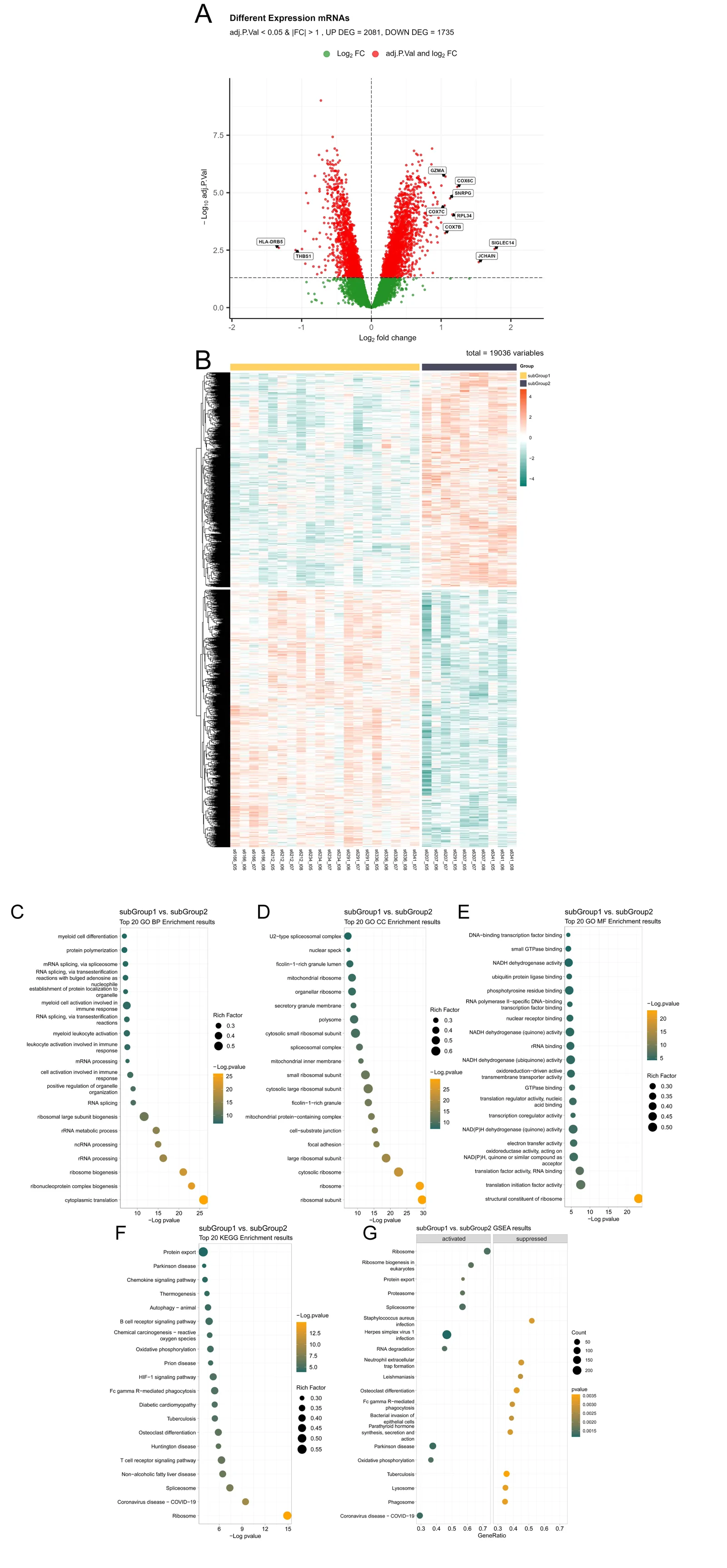
Identification of DEGs and their roles in sleep deprivation subtypes. (**A**) A volcano map showing DEGs between sleep deprivation subtypes. (**B**) A heatmap showing DEGs between sleep deprivation subtypes. (**C**) GO-BP enrichment analysis of DEGs. (**D**) GO-CC enrichment analysis of DEGs. (**E**) GO-MF enrichment analysis of DEGs. (**F**) KEGG enrichment analysis of DEGs. (**G**) GSEA showing pathways significantly related to sleep deprivation.

To further clarify differences in biological functions and pathways, we first compared the DEGs between the two subtypes (Fig. **8A** and **B**). Then, we performed GO and KEGG analyses (Fig. **8C**-**8F**, Supplementary Table **S5**). GSEA analysis showed that ribosome, ribosome biogenesis in eukaryotes, protein export, proteasome, spliceosome, herpes simplex virus 1 infection, RNA degradation, Parkinson disease, oxidative phosphorylation, and coronavirus disease-COVID-19 were activated, while staphylococcus aureus infection, neutrophil extracellular trap formation, Leishmaniasis, osteoclast differentiation, Fc gamma R-mediated phagocytosis, bacterial invasion of epithelial cells, parathyroid hormone synthesis, secretion and action, Tuberculosis, lysosome, and phagosome were suppressed (Fig. **8G**, Supplementary Table **S5**).

### 3.7 qPCR Demonstrated 6 Hub Immune Genes in the Auditory Cortex, Hippocampus, and Hypothalamus Tissues

**Fig. (9):**
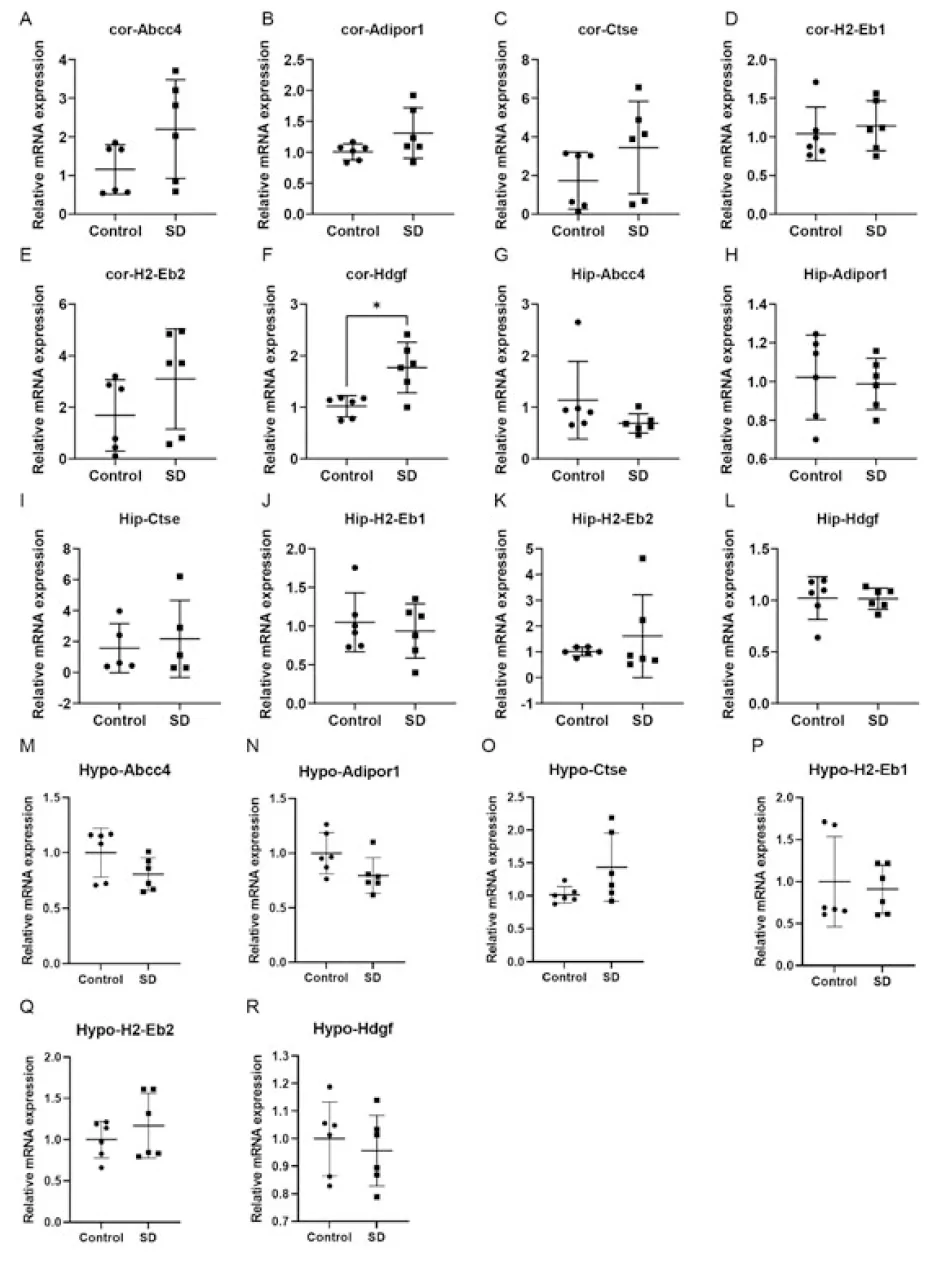
The expression of 6 hub immune genes was detected by qPCR in auditory cortex, hippocampus, and hypothalamus tissues after 3 weeks of sleep deprivation. Data are presented as means ± standard deviation, n = 6 for each group. * p < 0.05. SD, sleep deprivation.

We examined the expression of six hub immune genes in auditory cortex tissues after three weeks of sleep deprivation. The data showed that the level of Hdgf was significantly increased (Fig. **9A**-**9F**). In hippocampus and hypothalamus tissues, the expression of six hub immune genes was similar between the control and sleep deprivation groups (Fig. **9G**-**9R**). This finding provides limited but directionally consistent support for the candidate genes identified in the computational analysis, and their precise functional roles remain to be further elucidated through future research.

## 4 DISCUSSION

We conducted an integrated analysis of immune-related gene expression to elucidate the effects of sleep deprivation on biological functions and immune cell abundance, leveraging three publicly available microarray datasets (GSE98564, GSE98565, and GSE98566). Our findings provide novel insights into the molecular and cellular alterations associated with sleep deprivation, particularly highlighting disruptions in immune and hematopoietic processes, changes in key immune gene expression, and shifts in immune cell populations. By identifying specific gene modules and candidate immune genes, as well as characterizing molecular subtypes of sleep deprivation, this study deepens our understanding of the heterogeneity and complexity of the immune response to sleep loss.

Our results revealed a clear dysregulation of immune-related genes in sleep-deprived individuals, with 60 genes upregulated and 36 downregulated. Functional enrichment analyses consistently implicated these DEGs in biological processes central to immune regulation and hematopoiesis, including neutrophil migration, erythrocyte homeostasis, lymphocyte apoptotic regulation, and myeloid cell differentiation. This pattern of enrichment aligns with previous reports that sleep deprivation can perturb both innate and adaptive immune functions [7]. Our KEGG and GSEA analyses further confirmed the involvement of key immune pathways, such as T cell receptor signaling, Neutrophil Extracellular Trap (NET) formation, and the IL-17 signaling pathway, all of which have been implicated in immune modulation and in inflammatory processes exacerbated by sleep loss [28, 29].

A particularly notable finding was the alteration of immune cell abundances following sleep deprivation. CIBERSORT analysis demonstrated a significant reduction in the relative proportion of neutrophils in the sleep deprivation group, accompanied by increased proportions of naïve B cells and plasma cells. This observation requires careful interpretation: our transcriptome-based deconvolution reflects relative cellular composition within whole blood, not absolute circulating cell counts. Prior studies reporting neutrophilia following sleep deprivation predominantly measured acute stress responses *via* hormonal levels and hematological counts [30-32]. By contrast, chronic sleep restriction may trigger distinct compensatory mechanisms, including tissue redistribution of neutrophils, functional state transitions (*e.g*., enhanced margination or tissue infiltration), or long-term remodeling of hematopoietic output. Such tissue-specific dynamics could plausibly reduce neutrophil representation in peripheral blood transcriptomes despite systemic stress signals, representing a chronic adaptation rather than an acute stress response. The observed elevation in naïve B cells and plasma cells suggests a potential compensatory or dysregulated humoral response, potentially reflecting heightened antibody-mediated activity or altered B cell maturation under sleep-restricted conditions. These findings echo earlier reports that sleep modulates both cellular and humoral immune compartments, influencing not only lymphocyte subsets but also the quality and magnitude of antibody responses [33, 34].

To further dissect the molecular underpinnings of these immune changes, we performed WGCNA, identifying two gene modules (pink and red) most strongly associated with sleep deprivation. Through integration with immune-related genes, we identified five hub immune genes—HLA-DRB5, CTSE, ADIPOR1, HDGF, and ABCC4—as candidate markers potentially associated with sleep loss–induced immune alterations. Notably, qPCR validation revealed upregulation of Hdgf in auditory cortex tissues of sleep-deprived mice, suggesting a possible link between sleep deprivation and aberrant neuroimmune signaling or cellular proliferation in specific brain regions [35-39]. While previous studies have primarily focused on the role of HDGF in cancer biology and cell growth [40-42], its relevance in the context of sleep deprivation and neuroimmunology remains largely unexplored. Importantly, the functional role of these hub genes as direct regulators of sleep deprivation–induced immune changes is not yet established; they should be considered priority candidates for further mechanistic investigation rather than confirmed central regulators.

Importantly, our study went beyond conventional case- control comparisons by identifying two molecular subtypes of sleep deprivation, each characterized by distinct immune gene expression profiles and immune cell compositions. This molecular stratification mirrors emerging evidence of heterogeneity in sleep-related phenotypes and their immunological consequences [43, 44]. For example, gene set enrichment in one subtype indicated activation of pathways related to ribosome biogenesis, protein export, and viral infection response, whereas pathways associated with phagocytosis and NET formation were suppressed. These results underscore the existence of differential biological states or “endotypes” within sleep-deprived populations, which may underlie variability in susceptibility to infection, inflammation, or cognitive impairment observed clinically [45, 46]. From a translational perspective, these subtypes may represent distinct endotypes with differential clinical vulnerabilities: Subtype A (characterized by suppressed phagocytic/NET pathways and elevated B cell activity) might exhibit increased susceptibility to bacterial infections but preserved antiviral responses, whereas Subtype B (showing ribosomal activation and viral response enrichment) could manifest heightened inflammatory reactivity or altered vaccine responses. Such heterogeneity may partially explain why some individuals develop pronounced cognitive deficits or recurrent infections under sleep restriction while others remain relatively resilient. Future studies should prospectively link these transcriptomic subtypes to granular phenotypes—such as infection rates, inflammatory biomarkers, neurobehavioral performance trajectories, and vaccine responsiveness—to validate their clinical utility for risk stratification and personalized sleep interventions.

Our findings are consistent with and expand upon previous transcriptomic studies of sleep deprivation. Earlier work by Uyhelji *et al*. [20], utilizing the same GEO datasets, focused on temporal dynamics and transcription factor activity during sleep deprivation, reporting immune response genes among the top DEGs. However, our integrative approach specifically interrogated immune gene networks and immune cell signatures, providing a more granular view of sleep deprivation's impact on immune homeostasis. Similarly, animal studies by Lu *et al*. [47] and Narwade *et al*. [48] identified circadian and metabolic gene disruptions following sleep deprivation, but were limited to specific tissues and did not systematically analyze immune gene alterations across cell types or subtypes. Ruan *et al*. identified DEGs and key transcription factors in the mouse forebrain after six hours of sleep deprivation [49], but the results were not sufficiently convincing because of the small sample size (four control samples and six sleep deprivation samples). Laing *et al*. used a machine learning approach to analyze whole blood samples from people with sleep deprivation, but did not analyze the specific effects of sleep deprivation on immune function [50]. Our study addresses these gaps by leveraging large, multi-cohort blood transcriptomes and combining gene-centric and cell-centric analyses.

Despite these advances, several limitations should be noted. First, while the datasets analyzed were robust, our molecular subtype assignments were constrained by the relatively modest sample size within each subgroup. Larger, prospective cohorts are needed to validate these subtypes and their clinical relevance. Second, while qPCR verified the upregulation of Hdgf in the auditory cortex, additional *in vivo* and *in vitro* studies are required to determine whether the identified hub genes actively regulate or merely correlate with immune alterations in neural and peripheral tissues under sleep deprivation. Third, as our analysis was based on whole blood transcriptomes, tissue-specific immune responses—especially within the brain—may not be fully captured.

## CONCLUSION

Sleep deprivation was associated with coordinated changes in immune-related gene expression and inferred immune cell composition in whole blood, with findings most consistently implicating pathways relevant to innate immunity and hematopoiesis. The identified hub immune genes and molecular subtypes highlight heterogeneity in immune signatures among sleep-deprived samples and provide testable candidates for follow-up studies aimed at clarifying mechanisms and assessing their potential utility as biomarkers or intervention targets.

## Data Availability

The datasets generated and/or analyzed during the current study are available in the Gene Expression Omnibus (GEO) repository under accession numbers GSE98564, GSE98565, and GSE98566.

## LIST OF ABBREVIATIONS

SD: Sleep Deprivation
GO: Gene Ontology
KEGG: Kyoto Encyclopedia of Genes and Genomes
DEGs: Differentially Expressed Genes
DEIRGs: Differentially Expressed Immune-related Genes
WGCNA: Weighted Gene Co-expression Network Analysis
GEO: Gene Expression Omnibus
RMA: Robust Multiarray Average
adj.p: Adjusted p-value
IRGs: Immune-Related Genes
qPCR: Quantitative Polymerase Chain Reaction
PCA: Principal Component Analysis
GS: Gene Significance
MM: Module Membership
